# The PHD Finger of Human UHRF1 Reveals a New Subgroup of Unmethylated Histone H3 Tail Readers

**DOI:** 10.1371/journal.pone.0027599

**Published:** 2011-11-11

**Authors:** Nada Lallous, Pierre Legrand, Alastair G. McEwen, Santiago Ramón-Maiques, Jean-Pierre Samama, Catherine Birck

**Affiliations:** 1 Institut de Génétique et de Biologie Moléculaire et Cellulaire (IGBMC), Institut National de Santé et de Recherche Médicale (INSERM) U964/Centre National de Recherche Scientifique (CNRS) UMR 7104/Université de Strasbourg, Illkirch, France; 2 Synchrotron SOLEIL, Saint-Aubin, France; 3 Spanish National Cancer Research Centre (CNIO), Madrid, Spain; Consejo Superior de Investigaciones Cientificas, Spain

## Abstract

The human UHRF1 protein (ubiquitin-like containing PHD and RING finger domains 1) has emerged as a potential cancer target due to its implication in cell cycle regulation, maintenance of DNA methylation after replication and heterochromatin formation. UHRF1 functions as an adaptor protein that binds to histones and recruits histone modifying enzymes, like HDAC1 or G9a, which exert their action on chromatin. In this work, we show the binding specificity of the PHD finger of human UHRF1 (huUHRF1-PHD) towards unmodified histone H3 N-terminal tail using native gel electrophoresis and isothermal titration calorimetry. We report the molecular basis of this interaction by determining the crystal structure of huUHRF1-PHD in complex with the histone H3 N-terminal tail. The structure reveals a new mode of histone recognition involving an extra conserved zinc finger preceding the conventional PHD finger region. This additional zinc finger forms part of a large surface cavity that accommodates the side chain of the histone H3 lysine K4 (H3K4) regardless of its methylation state. Mutation of Q330, which specifically interacts with H3K4, to alanine has no effect on the binding, suggesting a loose interaction between huUHRF1-PHD and H3K4. On the other hand, the recognition appears to rely on histone H3R2, which fits snugly into a groove on the protein and makes tight interactions with the conserved aspartates D334 and D337. Indeed, a mutation of the former aspartate disrupts the formation of the complex, while mutating the latter decreases the binding affinity nine-fold.

## Introduction

**Figure 1:**
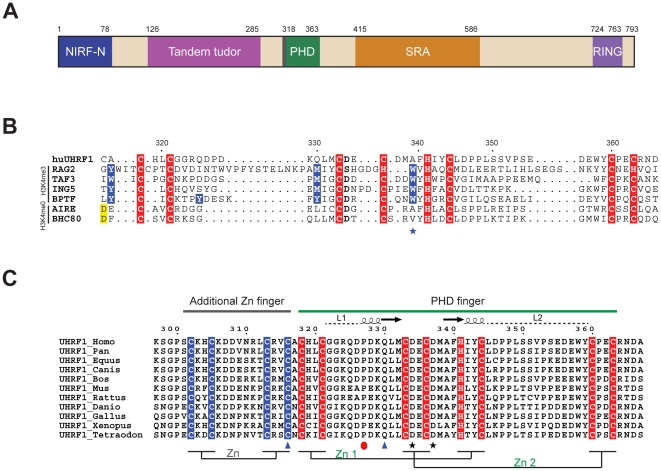
Domain architecture of human UHRF1 and sequence comparisons of its PHD finger. A- Representation of the multidomain protein huUHRF1, with each domain depicted in a different color. **B-** Sequence comparison of huUHRF1-PHD finger with other PHD fingers that recognize trimethylated or unmodified H3K4. Residues highlighted in blue, especially the tryptophan marked with a blue star, are implicated in trimethylated H3K4 recognition. The aspartate highlighted in yellow is key for recognition of unmodified H3K4. Residues highlighted in red are implicated in the C4HC3 coordination of the two zinc ions. **C-** Sequence alignment of the PHD region of the UHRF family shows the presence of a putative zinc finger composed by four invariant cysteines (in blue) preceding the canonical PHD finger. Within the PHD finger sequence, residues marked by blue triangles and black stars are implicated in histone H3K4 and H3R2 recognition, respectively. P327, which forces the bent conformation of the histone H3 peptide, is marked with a red circle.

Human UHRF1 (huUHRF1; ubiquitin-like containing PHD and RING finger domains 1), also called ICBP90, is a multi-domain nuclear protein associated with cellular proliferation and epigenetic regulation [1] (Fig. 1A). Through its SRA (SET and RING Associated) domain, UHRF1 recognizes hemimethylated DNA and targets DNA methyltransferase 1 (DNMT1) to hemimethylated replication forks [2], [3], [4], [5], [6], [7]. UHRF1 is up-regulated in various human cancers which may be related to its ability to sustain the transcriptional silencing of tumor suppressor genes by hypermethylation of their promoters [8], [9], [10]. Through its Tudor domain, UHRF1 recognizes the histone silencing mark H3K9me3 (histone H3 trimethylated at lysine 9), and exhibits stronger cooperative binding to H3K9me3-modified nucleosomes in the presence of CpG methylation [11]. UHRF1 is enriched in pericentric heterochromatin where it recruits different chromatin modifiers required for this chromatin replication [12], [13].

huUHRF1 contains a plant homeodomain or PHD finger (huUHRF1-PHD) that was shown to be necessary in the opening of dense chromocenter structures, possibly through its chromatin-binding ability [14]. Although huUHRF1-PHD was initially identified as a H3K9me3 binder [12], the crystal structure of the tandem tudor domain of this protein in complex with a histone H3K9me3 peptide and the recent characterization of the PHD finger showing no binding affinity for histones [15], [16] questioned again the exact role of huUHRF1-PHD. In the last five years, PHD fingers have emerged as protein motifs that specifically recognize histone H3 N-terminal tail. Two distinct subclasses of PHD fingers have been identified, which can specifically bind to either methylated (H3K4me, H3K9me) or unmethylated lysine residues on the histone H3 N-terminal tail with micromolar affinity. The recognition of the methylated lysine side chain requires an aromatic cage (an environment provided by a conserved tryptophan and additional aromatic residues), while the recognition of the unmodified lysine is mainly based on electrostatic interactions through a conserved aspartic acid [17], [18], [19], [20], [21], [22]. In most of the PHD finger structures recognizing unmodified or methylated H3K4, the arginine H3R2 fits snugly in a protein groove, where the positively charged guanidinium group forms a salt bridge with a conserved glutamate or aspartate.

In order to understand the role of huUHRF1-PHD we tested its ability to bind to peptides containing the N-terminal sequence of the histone H3 by native gel electrophoresis and we quantified the binding specificity by isothermal titration calorimetry (ITC). We proved that huUHRF1-PHD is a histone H3 reader that shows preference for unmethylated versus trimethylated H3K4. This binding is not influenced by the methylation state of H3K9 and requires the presence of the first two residues of the histone (H3A1 and H3R2). To determine the molecular basis of the interaction we solved the crystal structure of huUHRF1-PHD in complex with unmodified histone H3 peptide. Our results reveal a new mode of unmodified histone H3 recognition that involves an additional conserved zinc finger preceding the canonical PHD finger fold. By combining site-specific mutagenesis and ITC experiments, we show that the recognition of H3R2 is key for the interaction between huUHRF1-PHD and the histone H3, and that the methylation of H3K4 does not disrupt the interaction.

## Results and Discussion

### huUHRF1-PHD recognizes the unmodified N-terminal tail of histone H3

As an initial approach to characterize the PHD finger of huUHRF1, we compared the protein sequence with other PHD fingers whose specificity for binding either methylated or unmethylated H3K4 was well established both biochemically and structurally. A multiple sequence alignment showed that huUHRF1-PHD presents the pattern of cysteine and histidine residues that coordinate two zinc ions in the interlaced topology characteristic of the PHD finger fold. However, it lacks the conserved signatures for the recognition of either the methylated or the unmethylated H3K4 (Fig. 1B). Thus, based on sequence comparisons, the affiliation, if any, of huUHRF1-PHD to the two known subclasses of PHD fingers was not clear. Furthermore, a distinctive feature of huUHRF1-PHD is the presence of an 18 amino-acid segment preceding the canonical PHD finger region with four conserved cysteine residues in the proper disposition to form an additional putative zinc finger (Fig. 1C). Given the important differences with other PHD fingers, we explored the ability of huUHRF1-PHD to bind to histone H3 peptides.

**Figure 2:**
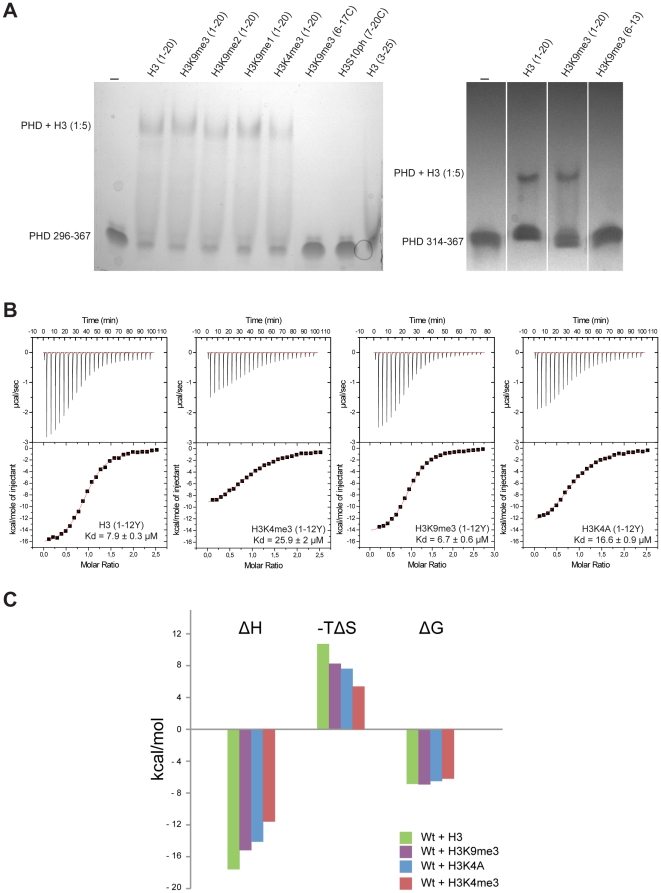
huUHRF1-PHD is a protein module that recognizes unmethylated histone H3. **A-** Characterization of the interaction between huUHRF1-PHD_296–367_ (left panel) or huUHRF1-PHD_314–367_ (right panel) with histone H3 peptides by native gel electrophoresis. At pH 9.4, UHRF1-PHD is negatively charged (pI = 5.14) and migrates into the gel, while the H3 peptides are positively charged and do not enter the gel. When the complex forms, the peptide retards the migration of the protein in the gel. The numbers in parenthesis indicate the length of the peptides corresponding to the histone H3 sequence. Sequence characteristics of the peptides are detailed in table S1. Different peptides with trimethylation of K4 (K4me3) or the three methylation states of K9 (K9me1, me2, me3), as well as phosphorylation of Ser10 (Sph10), were tested. **B-** Quantification of the interaction between the huUHRF1-PHD_296–367_ finger and different histone H3 peptides by ITC. **C-** Thermodynamic parameters of huUHRF1-PHD binding with different histone H3 peptides. The bar diagram shows the variation in the enthalpy (ΔH), entropy (-TΔS) and free energy (ΔG), as determined by ITC.

Two forms of the protein were produced, huUHRF1-PHD_314–367_, which contains the canonical PHD finger sequence, and huUHRF1-PHD_296–367_, a larger version that includes the additional putative zinc finger. We initially used native gel electrophoresis to test the interaction of the two proteins with peptides containing different fragments of the histone H3 sequence and bearing different post-translational modifications (Fig. 2A). Bands corresponding to complexes were observed by using H3 peptides covering residues 1 to 20, either without modifications or with trimethylated K4 or K9. Judging from the amount of protein shifted, it appears that the binding of huUHRF1-PHD_296–367_ to the peptides is stronger than the binding of the shorter construct. According to this experiment, we concluded that huUHRF1-PHD_296–367_ is able to recognize the histone H3 N-terminal tail *in vitro*, although the binding specificity for methylated versus non-methylated forms of the peptide was still unclear. Also, the affinity of the construct lacking the additional zinc finger towards H3 appears to be lower.

To clarify the binding specificity we performed ITC experiments to determine the dissociation constants (K_d_) of the interaction between huUHRF1-PHD and the histone H3 peptides (Fig. 2B). We showed that huUHRF1-PHD_296–367_ binds unmodified H3 with a K_d_ of 7.9 µM, an affinity value that is remarkably similar to that of AIRE1-PHD [17]. However, the affinity of huUHRF1-PHD_296–367_ for H3K4me3 decreased only 3-fold (K_d_ = 25.9 µM) while the interaction of AIRE1-PHD or BHC80-PHD with H3K4me3 was not detectable [17], [21]. The structures of the PHD fingers of AIRE1 and BHC80 show that the histone binding site can not accommodate the trimethylated side chain of H3K4 since it would result in steric hindrance. This appears not to be the case for huUHRF1-PHD_296–367_, which can still bind to H3K4me3, although with lower affinity. Surprisingly, huUHRF1-PHD_296–367_ is also able to interact with a H3 peptide in which H3K4 has been mutated to alanine (H3K4A) with an affinity that is only 2-fold lower than for H3 (K_d_ = 17 µM) but still higher than that for H3K4me3. The free energy of complex formation with the different H3 peptides is predominantly of enthalpic origin (ΔH = −17.6, −14.1, and −11.6 kcal/mol for H3K4me0, H3K4A and H3K4me3 respectively) and is partially compensated by a large and opposite entropy change (−TΔS = 10.7, 7.6, and 5.4 kcal/mol, respectively) (Fig. 2C). This thermodynamic signature is consistent with additional electrostatic and hydrogen bonds at the binding interface and with the perturbations of weak intermolecular interactions, including water molecules, and the conformational restrictions of side chains upon complex formation. The reduction in binding affinity with the different modified peptides is governed by changes in the enthalpic contributions, mainly due to the loss of hydrogen bonds involving the *ɛ*-amino group of H3K4, but is limited by the enthalpy/entropy compensation. These results indicate that the H3K4 residue weakly contributes to the affinity and that its binding pocket might be large enough to accommodate the trimethylated group. It also suggests that other histone residues may have a greater contribution to the binding affinity.

Despite previous studies that proposed the association of huUHRF1-PHD_296–367_ to H3K9me3, we discarded the importance of K9 for such interaction since the trimethylation of this residue did not change the dissociation constant significantly (K_d_ = 7 µM). On the other hand, we failed to detect binding to H3 peptides lacking the first two amino acids (Fig. 2A), and thus, we hypothesized that H3A1 and H3R2 might play a key role in the tight interaction of the histone with huUHRF1-PHD.

Unfortunately, the quantification of the apparent lower affinity of the shorter protein construct huUHRF1-PHD_314–367_ for H3 was unsuccessful due to the instability of the protein during the course of the ITC experiments.

### Crystal structure of huUHRF1-PHD in complex with H3

**Figure 3:**
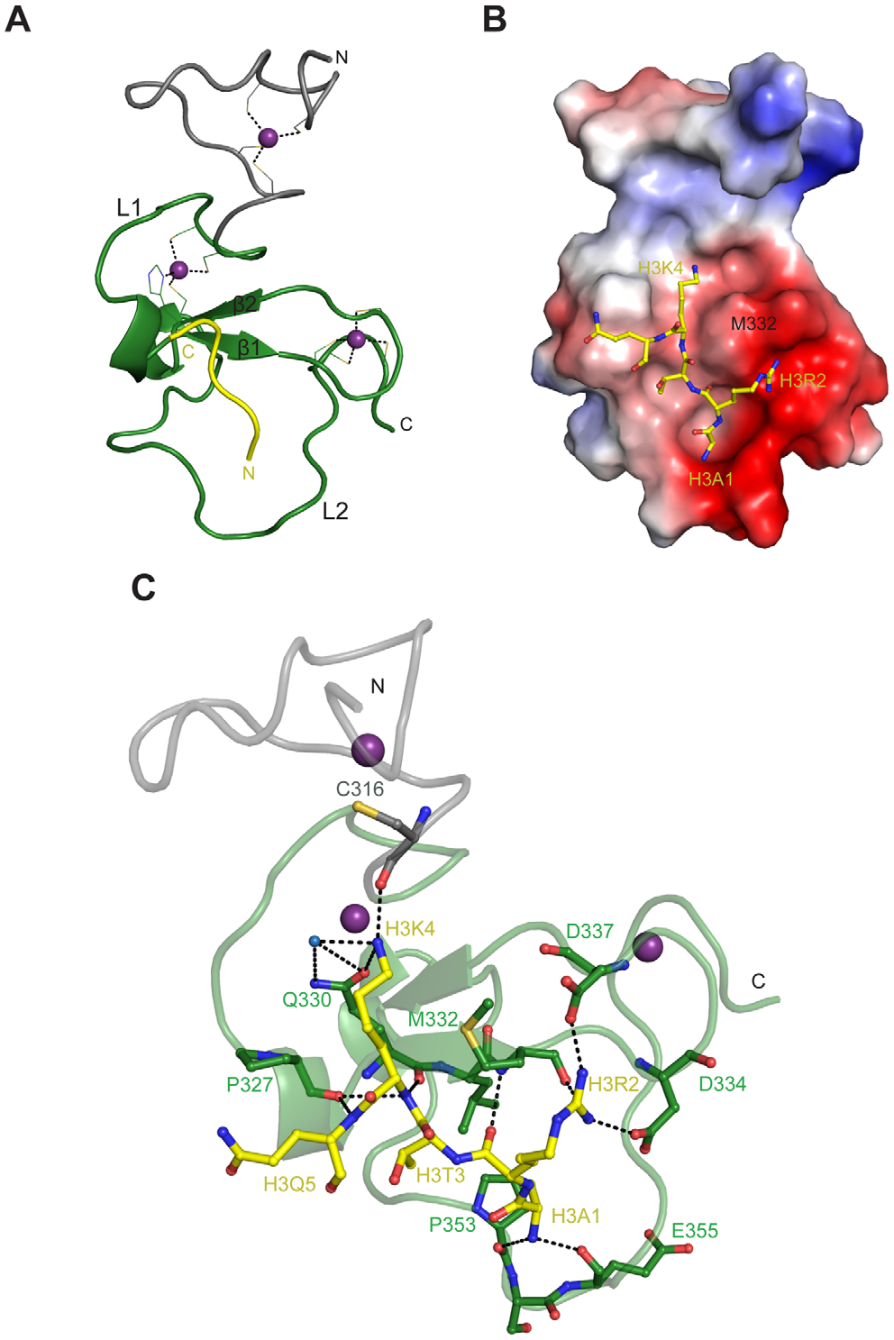
Crystal structure of huUHRF1-PHD_296–367_ in complex with unmodified histone H3 peptide. A- Cartoon representation of the crystal structure of huUHRF1-PHD_296–367_ (in green, with the additional N-terminal zinc finger depicted in grey) in complex with histone H3 peptide (in yellow). Residues coordinating the zinc ions (purple spheres) are represented as sticks. **B-** Electrostatic surface representation of huUHRF1-PHD_296–367_ with the bound H3 peptide shown as sticks. **C-** Detailed view of the association between huUHRF1-PHD_296–367_ and unmodified H3. The hydrogen bond and ionic interactions between the protein and the peptide are shown as dashed lines.

To determine the molecular basis of histone recognition, we solved the crystal structure of hUHRF1-PHD_296–367_ in complex with the unmodified histone H3 peptide at 1.95 Å resolution (Fig. 3) (Table 1). As predicted from the protein sequence, the structure consists of a canonical PHD finger (residues 318–363) preceded by an additional zinc finger (residues 296–317) (Fig. 3A). The PHD finger is formed by a pair of antiparallel β-strands (β1: 330-332, β2: 339–341) flanked by two short α-helices (residues 327–329 and 342–344) and two long loops, L1 and L2. The additional zinc finger involves the residues C302, C305, C313 and C316, which are strictly conserved across the UHRF1 family (Fig. 1C), and delimits the H3K4 binding site, as detailed below.

**Table 1:** Data collection, processing and refinement statistics.

Crystal	huUHRF1-PHD_296–363_ + H3 peptide	huUHRF1-PHD_314–363_
**Data collection and processing**		
Processing software	XDS/XSCALE	HKL2000
Synchrotron, beamline	SOLEIL, Proxima-1	SOLEIL, Proxima-1
Wavelength (Å)	1.284	0.977
Space group	*P*4_3_2_1_2	*P*6_5_22
Unit cell parameters a, b, c (Å)	42.30 42.30 182.70	51.01 51.01 83.63
Molecules/asymmetric unit	2	1
Resolution (Å)	41.21–195 (2.00–1.95)	24.39–1.45 (1.48–1.45)
Unique reflections	22,160	11,466
Redundancy	4.96 (4.7)	6.2 (2.3)
Completeness (%)	95.8 (83.9)	95.1 (67.8)
R_merge_	0.048 (0.528)	0.067 (0.314)
I/σ(I)	21.5 (3.1)	23.2 (3.0)
**Refinement**		
Refinement software	PHENIX	PHENIX
R_work_/R_free_	0.178/0.219	0.169/0.197
RMSD from ideal geometry, bonds (Å)	0.007	0.007
RMSD from ideal geometry, angles (°)	1.095	1.155
Mean B, protein (Å^2^)	44.1	35.1
Mean B, solvent (Å^2^)	47.7	46.2
Protein atoms	1181	554
Water molecules	87	55
Ramachandran plot:		
Most favoured regions (%)	97.67	98.44
Additionally allowed regions (%)	2.33	1.56
Disallowed regions (%)	0	0
PDB code	3ZVY	3ZVZ

Values in parentheses are for the highest resolution shell.

The histone peptide binds in a shallow groove on the protein surface, and only five out its eight residues were clearly observed in the electron density maps (Fig. 3B). The peptide does not adopt an extended β-strand conformation as was observed in the structures of AIRE1 or BHC80 [17], [18], [21], but instead it folds in a bent conformation with a sharp turn of nearly 90° at the position of H3K4, which projects its carbonyl group outwards (Fig. 3B,C). Interestingly, huUHRF1-PHD_296–367_ has a longer L1 loop forming a γ turn just before the β1 strand. This γ turn harbors P327, a residue which is strictly conserved in the UHRF1 family, and which prevents the extended conformation of H3 after K4 (Fig. 1C). A similar bent conformation was observed in the binding of H3K4me3 to the PHD of MLL1, where residue Y1581 plays the same role as P327 in huUHRF1 [23].

**Figure 4:**
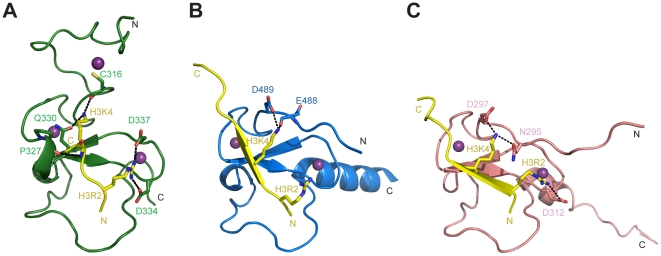
Structural comparison of PHD fingers recognizing the unmodified N-terminal tail of histone H3. The PHD fingers of huUHRF1-PHD_296–367_ (**A**), BHC80 (PDB: 2PUY) (**B**) and AIRE (PDB: 2KE1) (**C**) are shown in cartoon representation, with the bound H3 depicted in yellow.

huUHRF1-PHD_296–367_ accommodates the side chains of H3K4 and H3R2 simultaneously in two adjacent binding surfaces that are separated by the side chain of a conserved methionine (M332) (Fig. 3B,C). The side chain of H3K4 sits in a broad flat open surface, with the ε-amino group making hydrogen bonds with the side chain of Q330, with the carbonyl oxygen of C316 and with a water molecule that simultaneously binds to the side chain of Q330. Although methylation of H3K4 could hamper the interaction with C316 and Q330, the binding surface is large enough to accommodate the bulkier side chain without steric clashes. As already mentioned, in AIRE-PHD1 and BHC80 the side chain of H3K4 fits into a narrow channel with the side chains of two polar residues (D297 and N295 in AIRE and D489 and E488 in BHC80) interacting with the lysine ε-amino group (Fig. 4) [17], [18], [21]. As far as we know, huUHRF1-PHD_296–367_ provides the first example of a PHD finger where an aspartate residue is not involved in the recognition of the unmethylated H3K4.

As anticipated by the binding studies, the first two residues of the H3 peptide are important for the formation of the complex and are strongly anchored to the protein. The N-terminal amino group of H3A1 is hydrogen bonded to the carbonyl oxygen atoms of P353 and E355. On the other hand, the backbone carbonyl oxygen of H3R2 is hydrogen bonded to the amino group of M332, while its side chain sits in a pocket adjacent to the H3K4 binding site, with the guanidinium group interacting with the carboxylates of the invariant residues D334 and D337 (Fig. 1C).

It is noteworthy that C316 is part of the coordination sphere of the extra zinc ion found in the structure and simultaneously binds to the side chain of H3K4. Therefore, the formation of this additional zinc finger must be important for the correct orientation of C316 and for the recognition of H3K4. Indeed, we were unable to determine the structure of the complex between H3 peptide and huUHRF1-PHD_314–367_, the shorter form of the protein that lacks the additional zinc finger. Crystals of huUHRF1-PHD_314–367_ obtained in different crystallization conditions and grown in the presence of a large molar excess of H3 peptides belong to space group P6_5_22 and diffracted X-rays up to 1.43 Å resolution. However, the crystal structures invariably showed one protein molecule in the asymmetric unit with no peptide bound. The structure of huUHRF1-PHD_314–367_ in the free state is very similar to the structure of huUHRF1-PHD_296–367_ in complex with the histone H3 peptide except for a significant shift of residues 353–358 in loop L2, which anchor the N-terminal amino group of H3A1 in the huUHRF1-PHD_296–367_ -H3 structure, and a major conformational change of the N-terminal region (residues 314–316) (Fig. S1A). The Cα traces of residues 317–365 superimpose with an rmsd of 0.90 Å, which decreases to 0.60 Å if residues 353–358 are excluded. In the absence of the additional zinc finger, the N-terminal tail (residues 311–316) of huUHRF1-PHD_314–367_ blocks the binding of the histone peptide in two different ways. First, the Cα of C316 is displaced by 3.3 Å towards the histone H3 binding site and its side chain is flipped towards the position of the H3K4 side chain in the huUHRF1-PHD_296–367_-H3 structure. This conformation of C316 suggests that when this residue is not engaged in the additional zinc finger, it could interfere with histone binding. This hypothesis is supported by the apparent lower affinity of huUHRF1-PHD_314–367_ for H3 peptides observed by native gel electrophoresis. Second, due to crystal packing, the N-terminus of a symmetrically related molecule occupies the histone binding site (Fig. S1B). The residues involved in these strong lattice contacts (G311, H312 and M313) do not belong to the sequence of huUHRF1 but are carried over from the expression vector (Fig. S1C). All together, the flexibility of the C316 residue and the crystal packing artifact could explain the difficulty to obtain crystals of huUHRF1-PHD_314–367_ with the histone H3 peptide bound.

### Validation of key residues for H3 recognition

**Figure 5:**
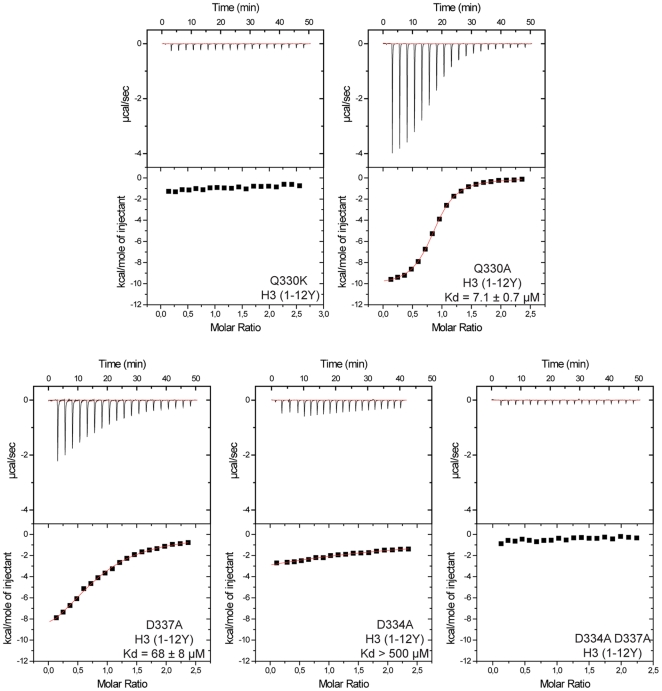
Characterization of key residues for the recognition of the histone H3 tail by huUHRF1-PHD_296–367_. ITC quantification of the effect of introducing point mutations at residue Q330, which interacts with H3K4, or at the two residues D334 and D337, that bind to H3R2. The K_d_ values for the mutants Q330K and D334AD337A could not be determined because no binding signal was detected with the H3 peptide.

The role of the residues involved in recognition of H3K4 and H3R2 was further analyzed by mutagenesis and ITC (Fig. 5). Q330, the residue interacting with the side chain of H3K4, was replaced by either an alanine (Q330A) or a lysine (Q330K). The mutation Q330A did not affect the affinity for H3 peptide (K_d_ = 7.1 µM), indicating that H3K4 is mainly recognized by the main chain carbonyl oxygen of C316, highlighting the implication of the additional zinc finger for histone recognition. On the other hand, we were unable to detect the interaction with Q330K, most likely due to the charge repulsion with the side chain of H3K4.

The two aspartates implicated in H3R2 recognition were also mutated to alanine. As expected from the tight interaction of these residues with H3R2, the mutations had a stronger effect on the binding. D337A increases the dissociation constant by approximately nine-fold (K_d_ ∼70 µM), while D334A disrupts the interaction (K_d_>500 µM), and the double mutant D334A/D337A shows no detectable binding. The stronger effect of D334A could be due to the fact that, in addition to binding to H3R2, the side chain of D334 stabilizes the loop L2 that anchors H3A1 via a hydrogen bond to the main chain N atom of W358. The presence of the aspartate D334 is highly conserved among the PHD fingers that recognize histone H3, while D337 is less well conserved.

These results show that the recognition of unmodified histone H3 by huUHRF1-PHD_296–367_ finger is mainly due to the interactions between the two aspartates, D334 and D337, with H3R2. Any alteration of these interactions strongly affects H3 recognition, while disrupting the interaction with H3K4 has a small effect.

### Structural and thermodynamic comparisons with other reported studies

During the submission of the present work, three independent studies have reported the interaction between huUHRF1-PHD and histone H3, determined structures of complexes, and quantified binding affinities. Superpositions of the current structure (PDB code 2ZVY) with the crystal structures determined by Rajakumara *et al.* [24] (PDB code 3SOU) and by Hu *et al*. [25] (PDB code 3SHB) give an average rmsd of 0.29 Å and 0.52 Å over 64 pairs of Cα atoms, respectively (Fig. S2A), showing that the crystal structures of huUHRF1-PHD are very similar. The superposition of the histone peptides bound in the different structures give an average rmsd of 0.10 Å over 4 pairs of Cα atoms (0.80 Å over 4 pairs of all atoms), validating the proposed model of interaction between huUHRF1-PHD and histone H3. The discrepancies between the three crystal structures and the model that Wang *et al.* [26] determined by NMR spectroscopy (PDB code 2LGG) are more important with an average rmsd of 3.70 Å over 64 pairs of Cα atoms (Fig. S2B). In agreement with our data, Hu *et al.,* and Rajakumara *et al*., showed that huUHRF1-PHD binds unmodified H3 peptide with a 3-fold higher affinity than for H3K4me3 peptide. However, we determined a K_d_ value of 7.9 µM for the unmodified H3 peptide which is 8.5 and 3.6-fold larger than those published by Hu *et al.* (0.93 µM) and by Rajakumara *et al.* (2.2 µM), respectively [24], [25]. The different values of the thermodynamic parameters may be related to the different experimental conditions described in each study, particularly the pH and the ionic strength of the binding buffer and the temperature that may explain the observed variations in K_d_ and enthalpy/entropy terms. By using tryptophan fluorescence measurements instead of ITC, Wang *et al.* observed a 7-fold increased affinity for unmethylated H3 versus H3K4me3 peptides, and the K_d_ value for unmodified H3 (3.8 µM) is 2-fold lower than our value [26]. Concerning the characterization of the mutations D334A and D337A in huUHRF1-PHD that affect the recognition of H3R2, the two other ITC studies agree with our results about the reduced binding affinity with the mutation D334A compared to the D337A mutation, although again there are differences in the absolute values of the K_d_. Most importantly, these other structural and thermodynamic analyses agree with our study about the relative importance of the residues in huUHRF1-PHD and histone H3 tail, which are involved at the binding interface.

### Concluding remarks

In the past years, the PHD finger has emerged as a versatile protein module for the recognition of the histone H3 tail [27]. In this work we show that the PHD finger of human UHRF1 represents a new mode of histone H3 recognition that requires the appendage of an additional zinc finger, relies mainly on the recognition of H3R2, and shows preference for unmethylated H3K4, although it can accommodate H3K4me3 without compromising the complex formation.

Like huUHRF1-PHD, most of the PHD fingers that recognize histone H3 rely on the interaction between the side chain of H3R2 and at least one conserved aspartate residue located at the end of strand β1 (Fig 1B). The importance of this interaction is supported either by the mutation of the aspartate residue [28], the mutation of H3R2 [24], [25] or the methylation of the guanidinium group of H3R2 [19], [29], all of which result in a reduction of the binding affinity for H3. In the case of UHRF1-PHD, mono- or dimethylation of H3R2 reduce the binding affinity by approximately 5- and 20-fold, respectively, whereas mutations of H3R2 or D347 to alanine cause a 30-fold drop in binding affinity [25]. In the case of AIRE-PHD, which also recognizes unmodified H3, the effect of H3R2 methylation was stronger than in UHRF1-PHD, with a 30-fold decreased affinity for the monomethylation of H3R2, whereas the dimethylation of H3R2 disrupted the interaction [17]. In the same family of H3 readers, the PHD finger of BHC80 may behave differently since its crystal structure with unmodified H3 showed that the side chain of H3R2 did not interact with the protein [21]. Therefore, in our opinion, the importance of the recent discoveries about huUHRF1-PHD do not only stem from the fact that this PHD finger is able to strongly interact with H3R2 (a characteristic found in other PHD fingers) but also from the low selectivity that it exhibits towards the methylation status of H3K4. To our knowledge, UHRF1-PHD is the first example of a PHD finger that presents similar binding affinities for different methylation states of H3K4. New data are needed to reveal whether the biological function of UHRF1-PHD finger might be as important as a permissive H3K4 reader than as a H3R2 reader.

These structural and biochemical data raise an important question about the functional advantage given to UHRF1 by its potentiality of binding to different histone H3 modifications and the molecular mechanisms underlying its activity in gene transcription. Rajakumara *et al.*, have pioneered the genome wide studies of UHRF1, identifying more than 3,000 genes which are up- or downregulated upon UHRF1 silencing [24]. The detailed analysis of two of these genes proved that their transcriptional repression by UHRF1 is driven by the recognition of H3R2 by the PHD domain within the promoter regions. Further studies should help to unveil the biological significance and the chronology of the interactions between domains in UHRF1 (PHD, SRA and Tudor) and the variety of epigenetic marks in their differential methylation states.

## Materials and Methods

### Protein preparation

Wild type and mutant constructs of the PHD_296–367_ and PHD_314–367_ of huUHRF1 protein were cloned by using the Gateway technology (Invitrogen) into the expression vector pHGGWA [30]. (His)_6_-GST-tagged proteins were expressed in *Escherichia coli* strain Rosetta 2 in LB media supplemented with 0.1 mM ZnCl_2_ by IPTG-induction for 5 h at 20°C. Cells were resuspended in 50 mM Tris-HCl pH 7, 150 mM NaCl, 5% glycerol, 10 mM imidazole, 25 µM ZnCl_2_, 0.5 mM phenylmethanesulfonyl fluoride (PMSF) and 5mM β-mercaptoethanol (BME). After sonication and centrifugation, the sample was first applied to a Ni^2+^ loaded HiTrap Chelating HP column (GE Healthcare) and the proteins were recovered by elution with a 10–250 mM imidazole gradient. The sample was dialysed overnight to remove the imidazole and the fusion tag was removed by a 24 h digestion with His-tagged TEV protease at 4°C. After passing the sample through a second Ni^2+^ affinity column to remove undigested protein as well as the TEV protease, the sample was concentrated and applied to a gel-filtration chromatography column (Superdex 75, GE Healthcare) equilibrated in 20 mM Tris pH 7, 150 mM NaCl, 0.5 mM Tris(2-carboxyethyl)phosphine (TCEP), 25 µM ZnCl_2_ and 0.1 mM PMSF. The final purification step comprised anion exchange chromatography (UnoQ, Bio-Rad) and proteins were eluted in a 30–500 mM NaCl gradient and dialysed overnight against gel-filtration buffer. During UHRF1-PHD_314–367_ purification, the buffers were not supplemented with ZnCl_2_ and there was no need for an anion exchange chromatography step after gel filtration since the protein was already pure.

### Native Blue Gel Electrophoresis

huUHRF1-PHD at a concentration of 88 µM was incubated for 2 h at 4°C with different histone H3 peptides (1–20) in a 1∶5 ratio. Protein alone and Protein-H3 peptide mixtures were loaded on a 6% native polyacrylamide gel at pH 9.4 (Tris-CAPS) and run for 30 to 40 min at 150 V in running buffer at pH 9.4 containing 60 mM Tris-HCl and 40 mM CAPS, while keeping the electrophoresis cuvette inside an ice bucket to maintain the low temperature. The displacement of the PHD finger by the histone peptides was visualized by Coomassie staining.

### Isothermal titration calorimetry

Measurements were carried out at 20°C on an ITC_200_ Microcal calorimeter (MicroCal). Histone peptides (1–12Y, 1.5–2 mM) were injected to a sample of either wild-type or mutant huUHRF1-PHD (0.12–0.18 mM) in a reaction buffer containing 20 mM Tris pH 7, 150 mM NaCl, 0.5 mM TCEP, 25 µM ZnCl_2_ and 0.1 mM PMSF. A typical titration consisted of 19 injections of 2 µl of peptide into 203 µl of the protein solution at time intervals of 150–300 s. Control experiments were performed under identical conditions to determine the dilution heat of the titrant peptide into buffer. Data were analyzed using the software ORIGIN 5.0 (OriginLab Corporation) and all the measurements were performed, at least, in duplicates.

### Crystallization, data collection and structure determination

To increase the success rate of crystallization studies, a short histone H3 peptide including residues 1–8 was used, according to the specificity of the recognition site determined by native gel electrophoresis and ITC studies. Crystals of huUHRF1-PHD_296–367_ complexed with histone H3 peptide were obtained at 17°C using the vapor diffusion method by mixing a protein solution at a concentration of 606 µM (in 20 mM Tris pH 7, 150 mM NaCl, 0.5 mM TCEP, 25 µM ZnCl_2_ and 0.1 mM PMSF) with a 10-fold excess of peptide with an equal volume of reservoir solution (0.1 M Tris pH 8.5, 0.2 M MgCl_2_, 30% PEG 4000). Crystals were cryoprotected with 15% MPD and flash frozen in liquid nitrogen. On the other hand crystals of UHRF1-PHD_314–367_ were obtained at 17°C by mixing a protein solution at 883 µM concentration (in 20 mM Tris pH 7, 150 mM NaCl, 0.5 mM TCEP and 0.1 mM PMSF) in presence of different ratios of H3 peptides with an equal volume of reservoir solution (2.2 M Na malonate pH 7, 0.2 M 1,6 hexandiol). Crystals were cryoprotected with 5% glycerol and flash frozen in liquid nitrogen. X-ray diffraction data were collected from a single crystal at the Proxima 1 beamline in Synchrotron SOLEIL at the absorption peak of Zn (l = 1.284 Å). The data sets were processed and scaled by using programs XDS/XSCALE or HKL-2000 [31], [32] and the positions of the zinc ions in the crystals were found by the SAD method by using the program SHELXD [33]. The experimental phases were calculated using the programs SOLVE [34] and RESOLVE [35]. The models were built manually using COOT [36] and the structures were refined by use of REFMAC [37] and PHENIX [38]. Figures were generated using PyMol (http://www.pymol.org/).

### Sequence alignment

Sequence comparisons were done using Multalin [39] and ESPRIPT 2.2 [40] programs.

### Structural data deposition

Atomic coordinates and structure factors for the reported crystal structures of hUHRF1-PHD_296–367_ in complex with histone H3 tail and huUHRF1-PHD_314–367_ in the free state have been deposited in the Protein Data Bank under accession codes 3ZVY and 3ZVZ, respectively.

## Supporting Information

Figure S1 **Crystal structure of huUHRF1-PHD_314-367_ in the free state.** **A-** Cartoon representation of the huUHRF1-PHD_314-367_ structure (pink) superimposed to the structure of huUHRF1-PHD_296–367_ (green) in complex with the histone H3 peptide (yellow). The protein residue C316 and the side chains of histone K4 and R2 are represented as sticks. The Zn ions are drawn as purple spheres. **B**- Lattice contacts of huUHRF1-PHD_314–367_ with a neighbor protein across a crystallographic 2-fold axis. The N-terminal region of one molecule enters the histone H3 binding site of the symmetrically related molecule. The histone H3 peptide observed in the huUHRF1-PHD_296–367_ – H3 complex is represented here in the equivalent position and colored in yellow. **C-** Detailed view of the interactions between the proteins in the crystal lattice. The hydrogen bond and ionic interactions between the proteins are depicted as dashed lines. Two grey ellipses indicate the positions occupied by the histone H3 residues R2 and K4.
(TIF)

Figure S2 **Structural comparison of huUHRF1-PHD models obtained from different independent studies.** **A–**C-alpha trace superposition of huUHRF1-H3 complex determined in the present work (in green, PDB code 3ZVY) with the crystallographic models reported by Hu *et al.* [25] (in orange, PDB code 3SHB) and by Rajakumara *et al.* [24] (in bluish, PDB code 3SOU). All the protein subunits present in each asymmetric unit were included in the superposition and being depicted in the same color. The three Zn ions embedded in the protein structure are represented as spheres and colored purple, while the fourth Zn ion that mediates the interaction between symmetry related protein subunit is colored in cyan. **B-** Superposition of the present complex structure (colored as in panel A) with the best representative NMR model reported by Wang *et al*. [26] (PDB code 2LGG) that is colored blue with the bound histone peptide depicted in magenta.
(TIF)

Table S1 **Sequences of the histone H3 peptides used in the binding assays.**
(DOC)
