# Supplementary figures and images for: The PHD Finger of Human UHRF1 Reveals a New Subgroup of Unmethylated Histone H3 Tail Readers
Source: PLoS One. 2011 Nov 11;6(11):e27599. doi: 10.1371/journal.pone.0027599 (PMC3214078; doi:10.1371/journal.pone.0027599)

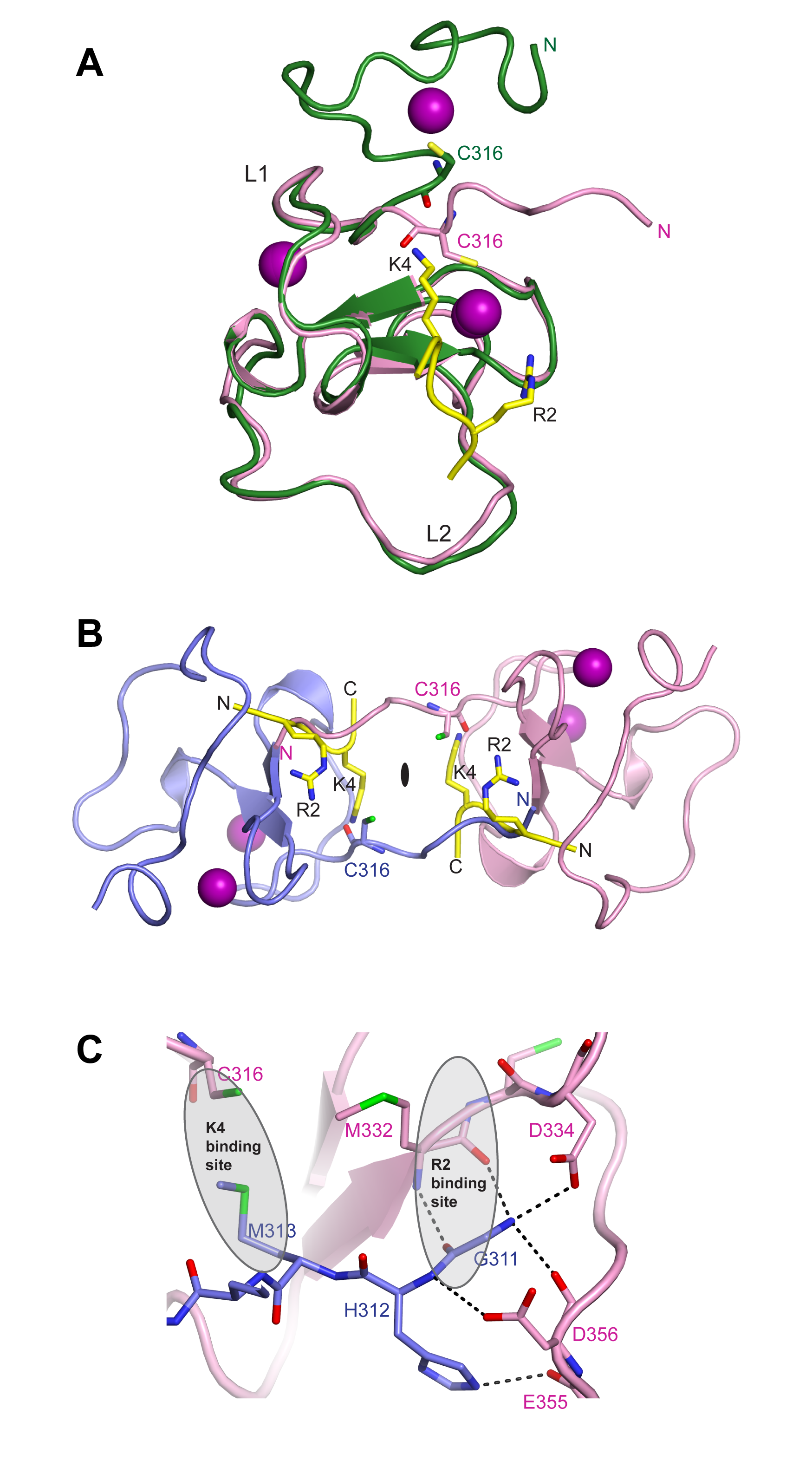

Supplement: Figure S1 — Crystal structure of huUHRF1-PHD314-367 in the free state. A- Cartoon representation of the huUHRF1-PHD314-367 structure (pink) superimposed to the structure of huUHRF1-PHD296–367 (green) in complex with the histone H3 peptide (yellow). The protein residue C316 and the side chains of histone K4 and R2 are represented as sticks. The Zn ions are drawn as purple spheres. B- Lattice contacts of huUHRF1-PHD314–367 with a neighbor protein across a crystallographic 2-fold axis. The N-terminal region of one molecule enters the histone H3 binding site of the symmetrically related molecule. The histone H3 peptide observed in the huUHRF1-PHD296–367 – H3 complex is represented here in the equivalent position and colored in yellow. C- Detailed view of the interactions between the proteins in the crystal lattice. The hydrogen bond and ionic interactions between the proteins are depicted as dashed lines. Two grey ellipses indicate the positions occupied by the histone H3 residues R2 and K4. (TIF) [file pone.0027599.s001.tif]

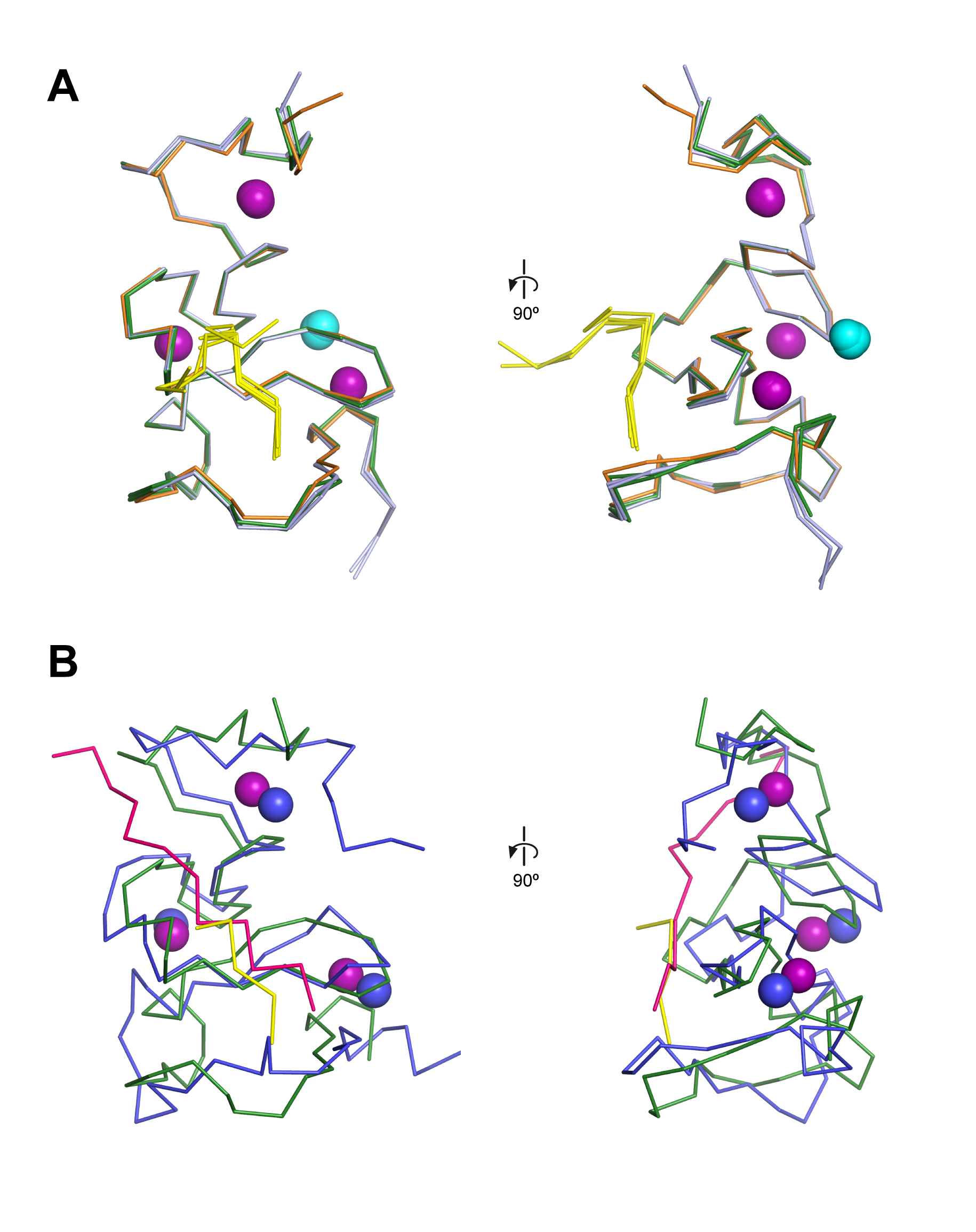

Supplement: Figure S2 — Structural comparison of huUHRF1-PHD models obtained from different independent studies. A–C-alpha trace superposition of huUHRF1-H3 complex determined in the present work (in green, PDB code 3ZVY) with the crystallographic models reported by Hu et al. [25] (in orange, PDB code 3SHB) and by Rajakumara et al. [24] (in bluish, PDB code 3SOU). All the protein subunits present in each asymmetric unit were included in the superposition and being depicted in the same color. The three Zn ions embedded in the protein structure are represented as spheres and colored purple, while the fourth Zn ion that mediates the interaction between symmetry related protein subunit is colored in cyan. B- Superposition of the present complex structure (colored as in panel A) with the best representative NMR model reported by Wang et al. [26] (PDB code 2LGG) that is colored blue with the bound histone peptide depicted in magenta. (TIF) [file pone.0027599.s002.tif]
