# Supplementary material for: The PHD Finger of Human UHRF1 Reveals a New Subgroup of Unmethylated Histone H3 Tail Readers
Source: PLoS One. 2011 Nov 11;6(11):e27599. doi: 10.1371/journal.pone.0027599 (PMC3214078; doi:10.1371/journal.pone.0027599)
Supplement: Table S1 — Sequences of the histone H3 peptides used in the binding assays. (DOC) [file pone.0027599.s003.doc]

| **Name** | **Peptide sequence** | **Native gel electrophoresis** | **ITC** |
| --- | --- | --- | --- |
| H3 (1-20) | ARTKQTARKSTGGKAPRKQL | ✓ |  |
| H3 (3-25) | TKQTARKSTGGKAPRKQLATKAA | ✓ |  |
| H3K4me3 (1-20) | ART**Kme3**QTARKSTGGKAPRKQL | ✓ |  |
| H3K9me1 (1-20) | ARTKQTAR**Kme1**STGGKAPRKQL | ✓ |  |
| H3K9me2 (1-20) | ARTKQTAR**Kme2**STGGKAPRKQL | ✓ |  |
| H3K9me3 (1-20) | ARTKQTAR**Kme3**STGGKAPRKQL | ✓ |  |
| H3K9me3 (6-17C) | TAR**Kme3**STGGKAPR**C** | ✓ |  |
| H3K9me3 (6-13) | TAR **Kme3**STGG | ✓ |  |
| H3S10ph (7-20C) | ARK**Sph**TGGKAPRKQL**C** | ✓ |  |
| H3 (1-12**Y**) | ARTKQTARKSTG**Y** |  | ✓ |
| H3K4me3 (1-12**Y**) | ART**Kme3**QTARKSTG**Y** |  | ✓ |
| H3K9me3 (1-12**Y**) | ARTKQTAR**Kme3**STG**Y** |  | ✓ |
| H3K4A (1-12**Y**) | ART**A**QTARKSTG**Y** |  | ✓ |

**Table S1**: Sequences of the histone H3 peptides used in the binding assays

The corresponding amino acid numbering is indicated in parentheses

Kme : methylated lysine

Sph : phosphorylated serine

**Y** : extra-tyrosine residue added to determine the peptide concentration by absorbance measurements at 280 nm.
